# Navajo Coal Combustion and Respiratory Health Near Shiprock, New Mexico

**DOI:** 10.1155/2010/260525

**Published:** 2010-06-30

**Authors:** Joseph E. Bunnell, Linda V. Garcia, Jill M. Furst, Harry Lerch, Ricardo A. Olea, Stephen E. Suitt, Allan Kolker

**Affiliations:** ^1^Department of the Interior, United States Geological Survey, 12201 Sunrise Valley Drive, Mail Stop 956, Reston, VA 20192, USA; ^2^Diné Environmental Institute, Diné College, 1228 Yucca Street, P.O. Box 580, Shiprock, NM 87420, USA; ^3^School of Public Health, George Washington University, 2300 I Street, NW, Washington, DC 20037, USA

## Abstract

Indoor air pollution has been identified as a major risk factor for acute and chronic respiratory diseases throughout the world. In the sovereign Navajo Nation, an American Indian reservation located in the Four Corners area of the USA, people burn coal in their homes for heat. To explore whether/how indoor coal combustion might contribute to poor respiratory health of residents, this study examined respiratory health data, identified household risk factors such as fuel and stove type and use, analyzed samples of locally used coal, and measured and characterized fine particulate airborne matter inside selected homes. In twenty-five percent of homes surveyed coal was burned in stoves not designed for that fuel, and indoor air quality was frequently found to be of a level to raise concerns. The average winter 24-hour PM_2.5_ concentration in 20 homes was 36.0 *μ*g/m^3^. This is the first time that PM_2.5_ has been quantified and characterized inside Navajo reservation residents' homes.

## 1. Introduction

### 1.1. Background

Within the United States (USA), a large group of people still use significant amounts of coal for home heating and some cooking. Among these are members of the Navajo Nation. Almost 175,000 people live within the boundaries of the sovereign Navajo Nation, an American Indian reservation located in the Four Corners area of Arizona, Colorado, New Mexico, and Utah [1]. The climate on the reservation is typical of American Southwest high deserts: hot in the summer, cold in the winter, with little precipitation and low humidity.

In 1957, the Navajo tribe and Utah International Inc. signed a contract for the mining of coal on the reservation. Utah International Inc. and Arizona Public Service Companies developed the Four Corners Power Plant adjacent to the mine, and coal deliveries to the first three units of the plant started in 1962. The total generating capacity of the Four Corners Power Plant is 2,040 megawatts. Located less than 10 km from the Four Corners plant is the 1,800 megawatt capacity San Juan Generating Station. The owners of this second coal-fired power plant in the Shiprock area include Power New Mexico, Tucson Electric Power, the Southern California Public Power Authority, and the City of Anaheim, California. If regarded as a single entity, the two plants are the second largest consumer of coal in the nation [2]. Most of the generated power is transmitted off the reservation by high-voltage lines to customers in Arizona cities, Las Vegas, Nevada, and even Los Angeles, California—over a thousand kilometers away [3]. 

Broken Hill Proprietary Minerals (BHP Billiton Limited) leases the coal mines from the Navajo Nation. As part of the lease agreement, BHP is required to provide coal for domestic use free of charge to Navajos who reside within a certain radius from the mine. The free coal comes from the Late Cretaceous Fruitland Formation and is relatively low rank (subbituminous to high-volatile bituminous). The Fruitland coal is lower in calorific value (10,646 +/− 1590 BTU/pound) and higher in ash content (22.24 +/− 7.02%) than coals found in other areas of the Navajo Nation, such as Black Mesa (10,910–11,560 BTU/pound, 4.7–9.1%) [2]. Many people who live in the eastern San Juan Basin (Shiprock area), despite having access to free coal from the Navajo mine, nevertheless choose to purchase Black Mesa coal at local flea markets due to its superior quality [2].

### 1.2. Shiprock and Respiratory Health

The Shiprock area is located in the northeastern part of the reservation; part of the area is in the San Juan River valley. Two features make the Shiprock area somewhat unique on the reservation: atmospheric thermal inversions trap air pollution low to the ground, especially in winter [2], and two large mine-mouth coal-fired power plants are located in the vicinity, with a third plant in the planning stages. Both existing power plants are exempt from regulation under the US Environmental Protection Agency 1990 Amendments to the Clean Air Act due to their age. Most residents of the Shiprock area obtain local coal at little or no cost. Compared to coal from other parts of the reservation, such as Black Mesa, Shiprock area coal tends to be lower in quality as measured by relative ash content and lower calorific value.

Because of thermal atmospheric inversions, often during winter a thick brown layer of smog hangs low over Shiprock, and many local residents attribute much if not all of that to the nearby power plants (e.g., [4]). As compared to other areas of the US with significant amounts of smog derived from vehicles, there are relatively many fewer automobiles, trucks, buses, tractors, and so forth, in Shiprock. The present study was undertaken to better understand the relationship between the perceived risk to respiratory health from ambient air quality versus the risk presented by indoor coal combustion. 

Compared to the general US population, American Indians suffer disproportionately from respiratory morbidity [5–9]. A variety of environmental risk factors have been associated with respiratory illness and chronic respiratory conditions in many general populations. Lower respiratory tract illnesses in infancy and respiratory symptoms in childhood are more common among children who have parents who smoke [10], have not been breastfed [11], or live in crowded conditions or use day care [12]. Both acute respiratory illnesses and chronic conditions such as asthma are significantly more common among lower income populations. Indoor and outdoor dust inhalation may also be a factor [13, 14]. However, relatively few of these risks appear to explain the excess in respiratory morbidity noted among the Navajo Nation residents. Moreover, it has been estimated that only approximately 4% of the Navajo people are regular smokers, roughly one-eighth the smoking rate among non-Navajos [15]. Worldwide, indoor air pollution is recognized as a major risk factor for respiratory morbidity, especially among cultures burning biomass for cooking and heating (e.g., [16, 17]).

Of the seven respiratory conditions/diseases analyzed in the hospital records in the present study, cough, wheeze, and asthma were all significantly associated with indoor coal combustion in a previous epidemiological investigation of children in China [18]. Another report found that susceptibility to bronchitis was significantly associated with ambient PM_2.5_ and polycyclic aromatic hydrocarbons (PAHs) among children in a former coal mining area in the Czech Republic with numerous large coal-fired power plants [18]. Interestingly, von Mutius et al. [19] noted a protective effect of in-home coal burning for children at risk of developing hay fever or other immunoglobulin E antibody-mediated allergies in southern Germany. However, that study also found that children from coal-burning homes were more likely to develop pneumonia.

Morris et al. [20] conducted a case-control study for respiratory tract illnesses (RTI) in the western part of the Navajo nation. Navajo children who were diagnosed with bronchitis or pneumonia were matched by age and sex with children seen for a well-child visit or for a minor health problem. Fifty-eight age- and sex-matched pairs of children were analyzed. Significant risk factors for development of RTI were use of a wood-burning stove in the house, recent respiratory illness exposure, family history of asthma, dirt floors, and lack of running water in the home. However, when all factors were entered into a multivariate model, only wood-burning stove use and respiratory illness exposure were independently associated with higher risks of RTI. This study supports the hypothesis that indoor air quality is a factor associated with respiratory illness among the Navajo. It is possible that wood was not the only fuel burned in those stoves, because many Navajos use mixtures of fuels, including coal, even in wood stoves. In a later study, Robin et al. [21] conducted a case control investigation of 45 Navajo children less than or equal to two years of age that considered risk factors related to acute lower respiratory-tract infection (LRI). These authors found that high indoor levels of fine particulate matter from wood burning inside the homes were associated with an increased risk of acute LRI (OR = 7.0).

Many Navajo Nation residents burn locally mined coal in their homes for heat, as this coal is the most economical energy source. Combustion of coal generates emission contaminants such as sulfur dioxide, carbon monoxide, nitrogen oxides, polycyclic aromatic hydrocarbons, and particulate matter containing trace metals [22–24]. The result is polluted air that may pose a health threat to residents. Additionally, the very young and the elderly spend more time indoors during winter when coal may be used for home heating, and people in these age groups tend to have compromised immunity relative to people in middle age (e.g., [25]).

In addition to burning coal inside their homes, residents of the Shiprock, New Mexico (NM), area are also exposed to ambient PM_2.5_ derived from the two nearby large coal-fired power plants. Construction of a third coal-fired power plant, Desert Rock, is scheduled to begin in the near future about 32 km southeast of Shiprock. The power plants near Shiprock produce noticeable amounts of smog, visible from miles away and often trapped low in the San Juan Basin by thermal atmospheric inversions. There are no other sizeable industrial activities in the area and relatively few motor vehicles. Although there are many other possible contributors to respiratory disease among residents in the area, power plant-derived air pollution is a likely contributor. However, the present research aims to address the issue of real versus perceived risk factors. Because it is so obviously visible, power plant smog is generally regarded by locals as one of the causes, if not the main cause, of respiratory disease—thus it is perceived as a primary risk factor. Although it may indeed be one risk factor, the real risk may in fact be greater from the indoor combustion of coal in nonoptimal stoves.

The objectives of the present study were to (1) examine aggregated respiratory data from the Northern Navajo Medical Center, an Indian Health Service (IHS) hospital in Shiprock, (2) document fuel and stove type and other household characteristics, (3) analyze samples of coal that are used locally, and (4) measure and chemically characterize airborne fine particulate matter (PM_2.5_), both ambient and inside selected residents' homes.

## 2. Materials and Methods

This study, including all survey instruments, procedures, and analyses employed, was approved by the Navajo Nation Division of Health Human Research Review Board, the Shiprock Chapter of the Navajo Nation, and the Navajo Nation Historic Preservation Department and was conducted with support from the Navajo Nation Environmental Protection Agency, the Indian Health Service (IHS), the Navajo Tribal Utility Authority, the Navajo Housing Authority, and the Bureau of Indian Affairs. All human subjects that participated in data collection signed statements acknowledging their informed consent. To ensure privacy and confidentiality of data when not being analyzed by authorized investigators, completed surveys were kept in a locked filing cabinet inside a locked office on the Diné College campus. Data remain the property of the Navajo people, and future access to the data must be authorized by the Navajo Nation Division of Health Human Research Review Board.

### 2.1. Household Exposure Risk Analysis

A seventeen-page survey instrument was designed in both the English and Navajo languages to obtain information about the physical nature of the homes, heating fuels used, and stoves, in order to assess factors that likely influence indoor PM_2.5_ exposure. Navajo-speaking student interns from Diné College in Shiprock, NM, were recruited to administer the surveys. Households were selected by professional and personal referrals forming a representative sample of convenience. Questions asked included such topics as the type(s) of fuel burned, age and condition of stoves, and household member behaviors such as smoking. Surveyed individuals were asked if they would consent to have PM_2.5_ monitoring conducted in their home at a later point, and a subset agreed. Linear correlation analysis was applied to this subset of data (*n* = 18) to test for any association between household characteristics predictive of PM_2.5_ concentration.

### 2.2. Hospital Records

Admission and outpatient visit data from April 1997 to December 2003 were obtained from the Northern Navajo Medical Center (NNMC) Indian Health Service (IHS) facility in Shiprock, NM, for the following seven conditions/diseases: asthma, bronchitis, chronic obstructive pulmonary disease (COPD), cough, pneumonia, upper respiratory tract infection (URI), and wheezing. The raw data for this analysis included the following information for each recorded instance: hospital admission date or date of outpatient service, location of patient's residence, and age and sex of patient. To protect privacy, all data were rendered anonymous and aggregated by NNMC staff so that no personal identifiers were released. Data organized by condition/disease included only the hospital admission date or date of outpatient service, the location of the patient's residence, and the patient's age and sex. The data did not differentiate or rank severity of outcome. All of the USGS-affiliated staff was trained to IHS standards to maintain confidentiality of records and for compliance with the Health Insurance Portability and Accountability Act (HIPAA) of 1996. 

The analysis was conducted in terms of an average annual prevalence, *p_ij_*, specific to the present research study, and is defined as,

(1) $$P_{ij}=\frac{100d_{ij}}{6.75r_{j}},$$

where *i* is one of the diseases, *j* is the patient place of residence, *d*_*ij*_ is the total number of patients from place *j* suffering disease *i*, and *r*_*j*_ is the number of Navajo residents in place *j*. Because the data were aggregated over six years and nine months, 6.75 was used in the denominator to estimate the annual prevalence rate. For spatial visualization and analysis, maps were created using ArcGIS (Environmental Systems Research Institute, Redlands, California, USA) software, with natural breaks grouping the data from each disease into five categories. Population data were obtained from the 2000 US Census and other sources (Table 1). Where the proportion of American Indians given in Table 1 is 100.0, it was possible to find the total Navajo population of the place directly, but it does not necessarily mean that everybody in the community is Navajo. 

### 2.3. Coal/Ash/Air Chemical Characterization

Two field sites were selected for repeated ambient PM_2.5_ sampling: site Nav78 and site Nav151. Site Nav78 was approximately 2 km from the Four Corners Power Plant, which burns coal from the open-cut Navajo mine operated by BHP Billiton Limited, and site Nav151 was approximately 10 km farther from the Four Corners Power Plant, at a lower elevation along the San Juan River Basin and which burns coal from an underground mine also owned by BHP. The sampling occurred over continuous 24-hour intervals with periodic adjustments to maintain a 16.7 L/min flow rate in order to maintain a 2.5 *μ*m cutoff. The filters were changed daily, at approximately noon at site Nav151 and 5:00 PM at site Nav78. Filters were removed from the filter packs indoors in a controlled environment using polytetrafluoroethylene-wrapped forceps (PTFE, e.g., Teflon).

#### 2.3.1. Organics

For analysis of organic material and inorganics (see below), PM_2.5_ samples were collected on 47 mm diameter PTFE filters with a 1.2 *μ*m cutoff (Pall Corporation, East Hills, New York, USA) in acid-cleaned PTFE filter packs (URG, Chapel Hill, North Carolina, USA). A cyclone (URG, Chapel Hill, North Carolina, USA) with D-50 cut point of 2.5 *μ*m at 16.7 L/min was attached to each filter pack with the inlet facing downward. A mass flow meter (Sierra Instruments Inc., Monterey, California, USA) was used to calibrate the flow rate. A ball-valve was used to regulate the 16.7 L/min requirement of the cyclone. A low-flow dry-gas meter (Hi-Q Environmental Products Company—SK-25, San Diego, California, USA) was used to record the total volume of air sampled. Because electricity was not available in the study area, 12 V DC Pumps (Rietschle/Thomas, Sheboygan, Wisconsin, USA) were powered by deep-cycle marine batteries, recharged nightly. All components of the filtration system were connected using 1/4 inch Tygon tubing. Plastic coverings were placed over the cyclone/filter pack and the marine batteries to protect them and prevent contamination. Field blanks were included in the sampling as well as laboratory blanks in the chemical characterization.

Sample filters were stored in 60 mL PTFE sample cups that had been precleaned with dichloromethane (DCM). On return to a USGS laboratory in Reston, Virginia, 25 mL of DCM was added to prevent microorganism activity and contamination. The PTFE sample cups were placed on a shaker for 16–18 hours to extract organic matter from the particles on the PTFE filters. After filtration, the liquid extract was transferred to a vial and reduced to a volume of 100 *μ*L under a flow of purified nitrogen. For gas chromatography/mass spectrometry (GC/MS) analysis, 2 *μ*L from each sample were injected into an HP 5973 Mass Selective Detector with an HP-5MS-fused silica column (30 m × 0.25 mm, 0.25 *μ*m film) (Hewlett-Packard/Agilent, Santa Clara, California) and run in a splitless mode. Helium was employed as the carrier gas with a consistent flow of 0.9 mL/min. The injector temperature was 260°C. The gas chromatograph oven temperature was programmed as follows: 50°C for 4 min, 50–150°C at 10°C/min, 150–230°C at 6°C/min, 230–300°C at 5°C/min, 300°C for 5 min. Hewlett Packard ChemStation software was used for instrumental operation and data collection. Database libraries, NIST 02 and Wiley 7.1, were used for identification of organic components. External standard polycyclic aromatic hydrocarbons (PAHs) of known identity and concentration were analyzed at the beginning of each suite of samples to determine response factors.

In addition to a comparison of all of the organic compounds identified in total ion current chromatograms, the following 26 PAHs were specifically examined: naphthalene, 1-ylnaphthalene, 2-methylnaphthalene, biphenyl, 2,6-dimethylnaphthalene, 2,6-dimethylnaphthalene, acenaphthene, 2,3,5-trimethylnaphthalene, fluorene, dibenzothiophene, phenanthrene, anthracene, 1-methylphenanthrene, 3,6-dimethylphenanthrene, fluoranthene, pyrene, benzo[a]anthracene, chrysene, benzo[b]fluoranthene, benzo[k]fluoranthene, benzo[e]pyrene, benzo[a]pyrene, perylene, indeno[1,2,3,cd]pyrene, dibenzo[ah]anthracene, and benzo[ghi]perylene. They were identified from the total ion current chromatograms on the basis of column retention time and selective ion monitoring.

Semiquantitative analysis was conducted by comparing the areas of the major ion from the samples with the areas of the major ions in PAH standard CUS-5734 (Ultra-Scientific, North Kingstown, Rhode Island, USA), which was diluted to a concentration of 0.5 ng/*μ*l for each of the 26 PAH compounds. The concentrations of the PAHs were determined by this comparison of areas and dilution factors were accounted for.

### 2.4. Trace Metals

Ambient PM_2.5_ sample collectors were constructed using a tripod stationary device (Hobo Weather Station—Onset Corporation, Bourne, Massachusetts, USA), with cyclone inlets positioned 2 m above ground level. See above for details concerning the rest of the sample collection apparatus. 

Filters were stored in 50 mm diameter polystyrene Petri dishes with lids that were acid-cleaned prior to use. In the first step of the cleaning procedure, tops and bottoms were placed in a dilute microsoap bath for fifteen minutes and rinsed with 18.2 mega-ohm/cm Milli-Q water. After being covered and dried overnight or dried in filtered air, the Petri dishes were placed in a 10% trace metal grade HNO_3_ bath, then rinsed with 18.2 mega-ohm/cm water, dried, and placed in clean plastic bags. 

At a USGS laboratory in Denver, Colorado, PTFE filters used for collection of inorganic trace elements were extracted in 1 mL of HCl and 3 mL of HNO_3_ and brought up to a 50 mL volume. The solution was analyzed for inorganic trace elements using inductively coupled plasma atomic emissions spectrometry (ICP-AES) model 3300DV (Perkin-Elmer) and inductively coupled plasma mass spectrometry (ICP-MS) ELAN 6000 (Perkin-Elmer/Sciex, Toronto, Ontario, Canada) for antimony, arsenic, beryllium, cadmium, chromium, cobalt, lead, manganese, and nickel; cold vapor atomic absorption (CVAA) for mercury; and hydride generation atomic absorption (HGAA) for selenium.

### 2.5. Assessment of Fine Particulate Matter Concentrations

For indoor PM_2.5_ quantification, personal DataRAM pDR1200 permissible real-time dust monitors with 2.5 *μ*m cutoff cyclones (Thermo Electron, Waltham, Massachusetts) and GilAir5 tri-mode air sampler pumps (Gilian/Sensidyne, Clearwater, Florida) run at approximately 4 L/min were used. These were powered either by alternating current using the home's electricity or by a standard car battery and direct current (DC) inverter. Monitors were run for 24 hours per sample.

## 3. Results

### 3.1. Household Exposure Risk Analysis

At time of publication, 137 interviews had been conducted in homes located in and around the town of Shiprock, NM. Not all participants answered all questions due to language difficulties or because of religious or cultural reasons. The majority of those surveyed used an indoor stove for heating (105 of 137, 77%); the remainder had electrical or other heating units. One quarter (34 of 137, 25%) of those surveyed were burning coal in stoves that were not designed to operate at the higher temperatures at which coal burns as compared to wood, and many of the stoves had visible cracks or were poorly ventilated to the outside. A similar fraction of the stoves were 10 years old or older (35 of 137, 26%). Most stoves had chimneys (106 of 117, 91%); however many chimneys had holes, cracks, and fissures. Although 21 of 136 (15%) of respondents reported themselves as smokers, that number includes those who smoke a local herb known as mountain tobacco only for ceremonial purposes, generally not more than several times per year. The complete responses from the survey instrument are available online [26].

Correlation analysis on the subset of surveyed homes for which PM_2.5_ data were available revealed only one variable that was statistically significant: controllable damper on the home's primary stove (*P* = .046). The variable with the next closest to significant association with fine particulate concentration was number of windows in the home (*P* = .301).

### 3.2. Hospital Records

**Figure 1:**
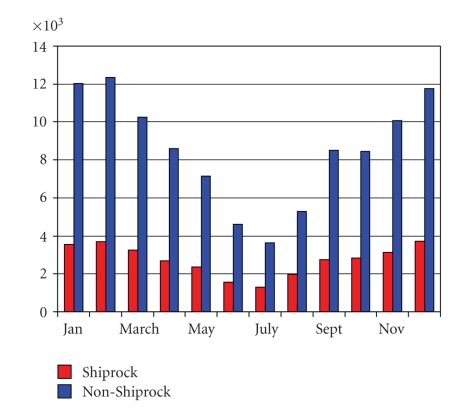
Total unadjusted raw numbers of hospital admissions/outpatient visits to NNMC for all seven diseases/conditions by month over the time period April 1997–December 2003, with residents of Shiprock in red and all other communities included in the study in blue.

The total number of records extracted for examination in this analysis was 133,759 over six years and nine months (April 1997–December 2003). This number includes all cases in the seven respiratory disease/condition categories that were seen at the NNMC. Cases that did not meet the following criteria were omitted from the analysis: ability to identify the locales on the Navajo Nation's official Department of Transportation map and availability of reliable population data for those communities. Because many Navajos who live off the reservation are seen at NNMC, cases from within a 20-mile buffer adjacent to the Indian Health Service's Shiprock Service Area boundary were included. This reduced the number to 116,719 for statistical analysis in 37 communities. There tended to be more hospital admissions and outpatient visits in the winter than in the summer (Figure 1), and the most commonly reported ailment was URI. 

**Figure 2:**
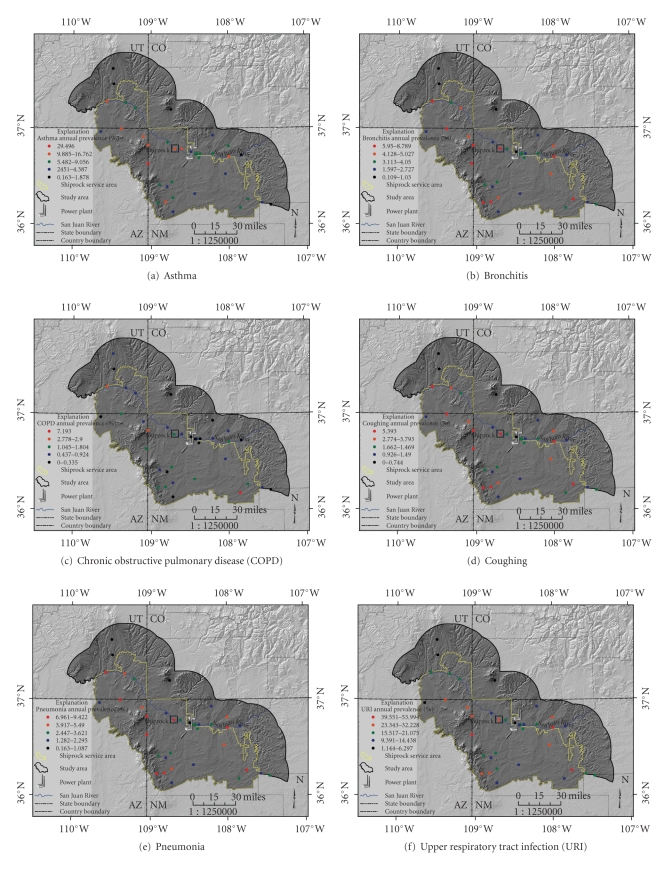

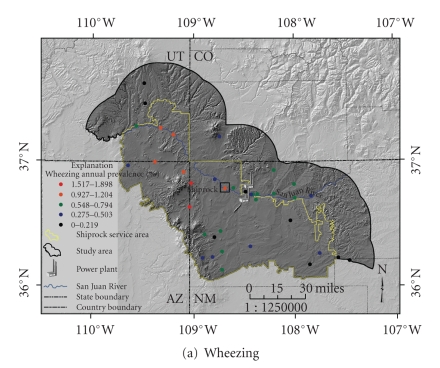
Geographic distribution of calculated annual prevalence during the time period April 1997—December 2003 in the Shiprock Service Area of the Navajo Nation, plus a 20-mile buffer to the north and east of the reservation boundary. UT:state of Utah; CO:state of Colorado; AZ:state of Arizona; NM:state of New Mexico. The town of Shiprock is highlighted, as are the locations of two coal-fired power plants. (a)–(g) display the following diseases/conditions, respectively: asthma, bronchitis, chronic obstructive pulmonary disorder (COPD), coughing, pneumonia, upper respiratory tract infection (URI), and wheezing.

Population-adjusted annual prevalence for the seven diseases/conditions for the 37 communities is presented in Table 2. Figures 2(a)–2(a) display the geographic distribution of the annual prevalence for each disease in the study area. In general, there appears to be an apparent spatial clustering of high prevalences to the west of Shiprock, although this is not uniformly the case.

### 3.3. Coal/Ash/Air Chemical Characterization

Ambient trace metal concentrations for antimony, arsenic, beryllium, cadmium, chromium, cobalt, lead, manganese, mercury, nickel, and selenium are displayed in Table 3. These elements were selected from the list of inorganic potentially hazardous air pollutants (HAPs) designated in the 1990 amendments to the Clean Air Act and subsequent publications. Concentrations are compared to health levels as defined by the Agency for Toxic Substances and Disease Registry (ATSDR) and the National Institute for Occupational Safety and Health (NIOSH) in the last column of Table 3. 

Seven coal samples were analyzed for the same eleven HAP trace metal concentrations (Table 4). These samples include BHP coal and Black Mesa coal as well as five samples taken from individual Navajo homes in the Shiprock area. These values, measured in parts per million on a whole-coal dry basis, are compared to the mean values for US coals (Table 4) to provide a reference for the concentrations. The only sample to exceed the US average values for any of the 11 HAPs was site Nav51, for lead.

#### 3.3.1. Organics

Fine particulate sample material was collected for organic chemical characterization at sites Nav78 and Nav151, indoors and outdoors, during the winter of 2006 and only once at site Nav151 during the summer of 2006. Each peak on the total ion current chromatogram generated in the analysis represents at least one distinct organic compound. The total number of peaks ranged from 7 to 131 per sample, with higher values indoors than outdoors and in winter than summer. The exact identity of the compound(s) corresponding to each peak is uncertain without comparison to known GC/MS standards. Standards were obtained for 26 PAHs in order to compare the relative amounts of these compounds per unit volume of air sampled because known health risks are associated with PAHs.

By this semiquantitative analysis, the PAH compound collected in greatest abundance from any of the samples was benzo[ghi]perylene at 116.1 ng (3.610 ng/m^3^ air) from the indoor site Nav151 during winter (Table 5). Only 11 of the 26 PAHs were detected and semiquantitatively analyzed for the 24-hour sample collected outdoors at site Nav151 in summer. This sample had the fewest number of PAHs (total mass 6.2 ng, or 0.193 ng/m^3^), although the outdoor winter Nav78 sample had the lowest total estimated mass of PAHs at 3.0 ng (0.093 ng/m^3^).

#### 3.3.2. Trace Metals

**Figure 3:**
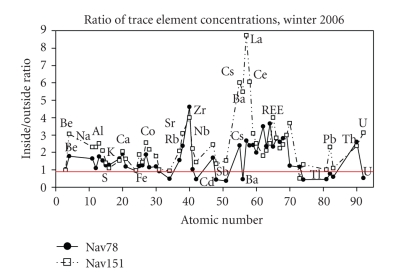
Ratio of indoor to outdoor trace element concentrations associated with PM_2.5_ at the two sites, 78 and 151, during winter 2006 when coal was burned indoors for heat. Elements falling above the 1 : 1 ratio line (in red) were detected at higher concentrations indoors relative to outdoors, while those falling between zero and one were detected at lower concentrations indoors relative to outdoors.

Two comparisons of indoor versus outdoor trace-element concentrations were made in February 2006, when coal was being used indoors, each based on 72-hour samples. The sites sampled are site Nav78, located within about 1.5 km of the power station, and site Nav151, located a much greater distance from the station. The ratios of indoor versus outdoor concentrations of elements extracted from the 72-hour filters are shown in Figure 3. For site Nav151, indoor concentrations were greater than or equal to outdoor concentrations (indoor/outdoor ≥1) for all elements except Se and Ta (Figure 3). Elements showing pronounced enrichment in indoor air (indoor/outdoor ratio ≥2.0) included Be, Na, Mg, Al, Si, Ca, Co, Ni, Rb, Sr, Zr, Nb, Ag, Cs, Ba, La, rare earth elements (REEs) (except Sm, in/out = 1.81), Pb, Th, and U. The most enriched indoor air concentrations, exceeding 3.0 times outdoor ambient air, were found for Be (indoor/outdoor = 3.07), Sr (in/out = 3.10), Zr (in/out = 3.99), Cs (in/out = 6.00), Ba (in/out = 5.49), La (in/out = 8.72), Ce (in/out = 6.06), Pr (in/out = 3.08), Tb (in/out = 4.00); Tm (in/out = 3.00), Yb (in/out = 3.67), and U (in/out = 3.13) (Figure 3). 

### 3.4. PM_2.5_ Indoor/Outdoor Data

Results were obtained from twenty indoor samples of at least 24 hours duration during the winters of 2005, 2006, and 2007. In ten other cases, the monitor did not run for the full 24-hour period, and those data were excluded. In some cases, the monitor or pump failed for some mechanical reason; for instance, the DC battery lost charge in one home without electricity. In other cases, the monitor was deliberately turned off either because it was deemed too loud, distracting, or annoying, or because of unspecified cultural/spiritual objection. In all but one of the twenty 24-hour samples, at least some coal was burned during the sampling period.

**Figure 4:**
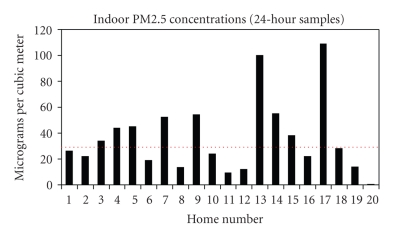
Average concentrations of winter (2005, 2006, and 2007) indoor PM_2.5_ over 24 hours at 20 homes. Home numbers 1–19 were burning coal during sampling period; home number 20 had an alternate heating source. Red dotted line indicates the 24-hour ambient US Environmental Protection Agency standard of 35 *μ*g/m^3^ for comparison.

**Figure 5:**
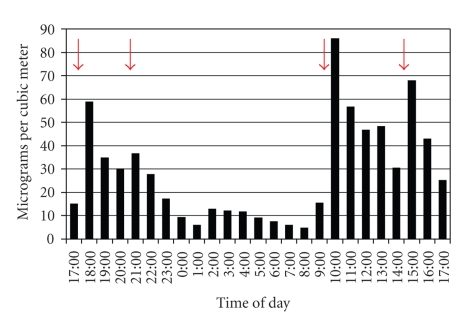
Representative display of PM_2.5_ concentration hourly averages over a twenty-four hour sampling period in one home (site Nav135). On the basis of interviews with residents, spikes in PM_2.5_ concentrations coincided with activities such as adding chunks of coal to the stove (red arrows).

The average 24-hour PM_2.5_ concentrations in winter of all 20 homes are presented in Figure 4. The overall average was 36.0 *μ*g/m^3^ (Figure 4). At the one home that had a propane heating source, the PM_2.5_ concentration was 0.293 *μ*g/m^3^, the lowest average value of all 20 samples. Excluding that sample, the average PM_2.5_ concentration of the 19 homes burning coal was 37.9 *μ*g/m^3^. The highest 24-hour average PM_2.5_ concentration was 109 *μ*g/m^3^, more than three times the EPA 24-hour ambient standard of 35 *μ*g/m^3^. There were large variations of PM_2.5_ concentration during the sampling periods, as demonstrated by the hourly averages illustrated by one representative sample (Figure 5).

Eight indoor 24-hour PM_2.5_ samples were collected during the summer of 2006. The overall average PM_2.5_ concentration of these eight homes was 12.0 *μ*g/m^3^. No coal was burned in these homes during the sampling period, although residents in one home reported cigarette smoking. 

Ambient 24-hour PM_2.5_ samples were taken at two locations, site Nav78 and site Nav151, in the summer of 2005. Over a six-day period, the overall average 24-hour PM_2.5_ concentrations at sites Nav78 and Nav151 were 13.70 *μ*g/m^3^ and 9.43 *μ*g/m^3^, respectively. In the winter of 2006, during the same 24-hour period when one of the indoor samples was being collected, a monitor was placed outside one of the homes. 

The average PM_2.5_ concentration of this sample was 9.95 *μ*g/m^3^. For comparison, the yearly 24-hour average ambient PM_2.5_ concentrations at the US Environmental Protection Agency monitoring station nearest these Shiprock homes in Farmington, NM, were 15, 21, 16, 15, and 11 *μ*g/m^3^ in 2001, 2002, 2003, 2004, and 2005 (the most recent data available at publication time), respectively.

## 4. Discussion

Here we present results from a multicomponent study addressing various aspects of real and perceived exposures to and risks of coal combustion products as relates to respiratory health in the Navajo Nation. We found that the respiratory disease burden (as measured by hospital admissions and outpatient visits to the NNMC for the seven disease/conditions noted) is increased in the winter as compared to summer (Figure 1), and yet the power plant emissions are greater in the summer than those in the winter (e.g., [27]). The likely reason for this increase in emissions is because the customers of the electricity generated by the Four Corners area power plants predominantly reside in cities such as Las Vegas, Phoenix, and Los Angeles, where demand is higher in summer, presumably for air conditioning. This may be offset somewhat by the higher probability of thermal inversions in winter rather than summer but is still suggestive that perhaps more than just the power plant smog is responsible for the higher number of cases in winter. Indeed, that respiratory diseases tend to occur more frequently in winter among the general public is well known.

Thermal atmospheric inversions exert the same effect on combustion products regardless of whether their generation is industrial or residential. The population center of Shiprock, situated low in the valley along the San Juan River, is affected by inversions more than the outlying communities, which are typically higher in elevation. Even though the power plant emissions are greater in the summer (e.g., [27]), smoke from residential chimneys is likely a significant factor for the observation of heavier air pollution in Shiprock than surrounding locales in winter when domestic coal combustion increases. 

Results of the stove/fuel-type/use survey indicated that household characteristics were rarely predictive of PM_2.5_ concentrations for that subset of 18 for which air monitoring was also conducted. Of the two most statistically significant variables that did have an association with indoor PM_2.5_ concentrations, number of windows seems to reflect that the better homes were ventilated, the lower the fine particulate concentrations measured (higher number of windows correlated negatively to higher concentrations of PM_2.5_). For the other variable, a controllable damper on a coal stove correlated to higher PM_2.5_ concentrations in the home, whereas PM_2.5_ levels were lower in homes with dampers that had no controls. Most likely, residents desiring to allow as little heat as possible to escape through the chimney tended to close controllable dampers at least partially, thus allowing more PM_2.5_ to enter living quarters.

In the present study, even in homes where stoves and/or chimneys had visible cracks, visible smoke rarely entered the rooms while coal was being burned. Residents might be more inclined to seal their stoves and chimneys better if they observed smoke coming into the home. As an indication that exposure to fine particulate matter has occurred even in the absence of plumes of smoke entering the living space, evidence of soot, sometimes quite heavy, was observed on walls and other surfaces. While in the present study nonairborne household soot was not analyzed, a number of mutagenic PAHs have been identified from soot in homes that burn coal in China [28].

Upon examination of the geographic distribution of the hospital records data (Figures 2(a)–2(a)), however, there is no apparent clustering of high prevalence in communities along the San Juan River Valley. There is, however, an apparent cluster of communities that rank high for most of the diseases/conditions west of Shiprock, ranging from the San Juan River at the northwestern boundary of the Shiprock Service Area to the southwestern boundary of the Shiprock Service Area. The town of Shiprock was ranked in the top ten for all seven of the diseases/conditions: of all 37 communities, Shiprock ranked #2 for asthma, #3 for coughing and wheezing, #6 for pneumonia, #7 for URI, #8 for bronchitis, and #9 for COPD. Interestingly, Red Valley—west-southwest of Shiprock—ranked #1 for five of the seven diseases/conditions (Kimbeto was highest for COPD, and Nageezi for URI; Red Valley ranked #6 and #3, resp.). Red Valley, southwest of Shiprock, is not in the San Juan River Valley, but it is lower in elevation than Shiprock, and, according to anecdotal evidence, power plant emission plumes often flow locally in that direction, especially during thermal atmospheric inversions.

For the first time, fine particulate matter from inside Navajo Nation residents' homes has been quantified and chemically characterized. Many residents of this region are exposed to PM_2.5_ inside their homes, at least in the winter, by the combustion of coal as a home heating fuel. The overall average 24-hour PM_2.5_ concentration inside 20 homes where coal was burned for heat exceeded the US Environmental Protection Agency 24-hour standard for ambient PM_2.5_ of 35 *μ*g/m^3^. This value provides a reference only, as there is no regulatory standard for indoor PM_2.5_. The present research has also documented that fully one quarter of the stoves used for coal combustion in this study were not designed to operate properly at the higher temperatures at which coal burns, as opposed to other fuels, and in many instances the stoves are in states of disrepair.

As for the two field sites selected for repeated ambient PM_2.5_ sampling during the summer of 2005, site Nav78 (average 24-hour PM_2.5_ concentration = 13.70 *μ*g/m^3^) was located significantly closer to the power plant and the Navajo mine. Thus, this site was exposed to a variety of PM_2.5_ sources, such as emissions from the stacks, coal dust from the actual mining of the coal, traffic on adjacent unpaved roads, fly ash, and the unloading/dumping of the coal from the mine train to the power plant (which creates large visible black smoke clouds). In addition, this site was possibly exposed to relatively less emissions from the stacks because the combustion byproducts are being released at least a hundred meters above ground. 

Site Nav151 (average 24-hour PM_2.5_ concentration = 9.43 *μ*g/m^3^) was farther away from the power plant, lower in elevation in the San Juan valley, and thus may have been exposed to a relatively greater fraction of the PM_2.5_ from the combustion of coal, emitted both from power plants and from people's chimneys, which accumulated lower in the valley and was trapped by thermal inversions. Site Nav151 was not near any major paved roads, and the only traffic on the closest unpaved roads consisted of the residents driving in or out of the site once or several times per day. 

Indoor and ambient 24-hour average PM_2.5_ concentrations were also measured at these two locations in the winter of 2006. The indoor values at sites Nav78 and Nav151 were 52.24 *μ*g/m^3^ and 18.90 *μ*g/m^3^, while outdoor values were 9.95 *μ*g/m^3^ and 13.04 *μ*g/m^3^, respectively. Curiously, those winter ambient PM_2.5_ concentrations at the two sites were almost the reverse of those measured in the summer, when the power plant emissions were somewhat higher: that is, the ambient PM_2.5_ concentration at the site right next to the power plant was 13.70 *μ*g/m^3^ in the summer and 9.95 *μ*g/m^3^ in the winter, while at the site down in the valley it was 9.43 *μ*g/m^3^ in the summer and 13.04 *μ*g/m^3^ in the winter.

The comparison of both organic compounds and trace elements indoors versus outdoors suggests that indoor coal use increases human exposure to both and may be a contributing factor to Navajo respiratory health problems. For example, site Nav78 was observed to contain overall trace element enrichment in the indoor air, but compared to site Nav151, a greater number of elements (Se, Mo, Cd, Sb, Ba, W, Tl, Pb, Bi, and U) show the reverse trend and are more concentrated in the outdoor filters. Elements showing this reverse enrichment are heavy elements that would appear to suggest a coal or coal-ash source, or another metal-enriched source, rather than an aluminosilicate source as from local road dust. 

All of the trace metal concentrations in ambient air in 2005 were detected at levels below the health level guidelines set by the ATSDR and NIOSH; however, these guidelines are intended to serve only as a reference because 24-hour ambient air standards do not exist. The majority of these standards are based on limitations set by the Occupational Safety and Health Administration [29]. Thus, these standards are intended for occupational settings, not for general ambient air quality. 

In terms of trace element concentrations and traditional measures of coal quality (e.g., ash content, BTUs, etc.) (data not shown), there was no significant difference among the samples taken from residences or the mine-mouth power plant, although the Four Corners coal was characterized by higher ash content than that from Black Mesa. Thus, on the basis of the samples analyzed, there appears to be no merit to the perception by some that inferior coal is made available to the people while “good” coal is reserved for the power plant.

Results for site Nav78 suggest that for the heavy elements analyzed, ambient exposure may outweigh exposure resulting from indoor coal use. However, at both locations, there were greater numbers of organic compounds indoors as opposed to outdoors. Because coal combustion is not the only source of organics indoors, future analyses may be focused on identifying these compounds. For instance, higher levels of heavy molecular weight PAHs could be indicative of coal combustion. 

Future studies that will directly link indoor PM_2.5_ concentrations and respiratory health outcomes in coal burning versus non-coal burning households in the Shiprock area are needed. The suggestion of a spatial pattern to poor respiratory health west of Shiprock also merits additional investigation. One expedient improvement to indoor air quality in Navajo homes would be to upgrade or exchange old stoves or those inappropriate for burning coal. Given economic realities on the Reservation, such action would likely necessitate intervention by an entity such as a large corporation or a philanthropic foundation.

## 5. Conclusions

This report presents the first systematic study of coal combustion's likely impacts on respiratory health in Shiprock area of the Navajo Nation. Over 130 homes in the Shiprock area were surveyed, and stoves in one-quarter of those homes were found to be inappropriate for coal combustion, even though residents there were burning coal. On the basis of spatial analysis of a robust set of hospital records data, residents of Shiprock and nearby communities appear to be at greater risk for respiratory disease than people in other communities not subject to thermal inversions, such as is typical for most of the Reservation. The presence of two large coal-fired power plants near Shiprock may contribute to that risk, but results from this study suggest that the risk could be reduced by making relatively simple and inexpensive changes to methods of home heating. Future studies to further examine the apparent link between coal combustion product exposure and respiratory health outcomes would be of benefit. While ambient air quality remains a concern, respiratory disease burden in the Shiprock area may be reduced by changing indoor home heating behavior and improving stove quality.

## Figures and Tables

**Table 1:** Sources of population figures used to calculate annual prevalence of seven respiratory diseases/conditions in the Shiprock Service Area (plus 20 miles off the reservation) of the Navajo Nation. An asterisk denotes a value based on a neighboring community.

Place	Population	% Navajo	Source
1. Aneth, UT	598	98.83	2000 Census
2. Aztec, NM	6378	9.31	2000 Census
3. Beclabito, NM	339	97.94	2000 Census
4. Blanco, NM	691	15*	2000 Census
5. Blanding, UT	3162	28.94	2000 Census
6. Bloomfield, NM	6417	16.71	2000 Census
7. Bluff, UT	320	35.01	2000 Census
8. Burnham Chapter, NM	240	99.58	http://www.nndes.org/
9. Counselor, NM	300	92.01	http://www.city-data.com/
10. Cudei, NM	621	95*	http://www.fallingrain.com/
11. Farmington, NM	37844	16.96	2000 Census
12. Fruitland, NM	5086	48.93	http://www.zipskinny.com/
13. Hogback Chapter, NM	1386	99.21	http://www.nndes.org/
14. Huerfano, NM	2366	97.1	http://www.nndes.org/
15. Kimbeto, NM	182	95*	http://www.fallingrain.com/
16. Kirtland, NM	6190	48.93	2000 Census
17. La Plata, NM	1021	10*	http://www.zipskinny.com/
18. Little Water, NM	571	99.3*	http://www.nndes.org/
19. Lybrook, NM	400	92.01	http://www.city-data.com/
20. Mexican Water, AZ	815	99.02	http://www.nndes.org/
21. Montezuma Creek, UT	507	96.06	2000 Census
22. Nageezi, NM	968	100.0	http://www.sjcounty.net/
23. Nenahnezad, NM	726	97.52	2000 Census
24. Newcomb, NM	705	100.0	http://www.sjcounty.net/
25. Ojo Amarillo, NM	829	95.54	2000 Census
26. Red Mesa, AZ	363	95*	http://www.city-data.com/
27. Red Valley Chapter, NM	468	95.09	http://www.nndes.org/
28. Sanostee, NM	1908	100.0	http://www.navajobusiness/
29 Sheep Springs, NM	821	96.2	http://www.nndes.org/
30. Shiprock, NM	8156	96.74	2000 Census
31. Teec Nos Pos, AZ	1323	96.0	http://www.nndes.org/
32. Toadlena, NM	442	100	http://www.sjcounty.net/
33. Tocito, NM	252	90*	http://www.fallingrain.com/
34. Towaoc, CO	1097	94.44	2000 Census
35. Two Grey Hills, NM	610	100.0	http://www.sjcounty.net/
36 Waterflow, NM	1606	78.0	http://www.househunterhq.com/
37. White Mesa, UT	277	98.19	2000 Census

**Table 2:** Annual prevalence (%) of seven respiratory diseases/conditions in the Shiprock Service Area from April 1997 to December 2003 among Navajo residents seen at the Northern Navajo Medical Center Indian Health Service hospital.

Community	asthma	bronchitis	COPD	coughing	pneumonia	URI	wheezing
Aneth, UT	8.824	4.337	0.577	2.056	5.49	18.5	0.927
Aztec, NM	8.779	4.539	0.524	2.469	2.295	29.505	0.599
Beclabito, NM	12.271	7.497	2.9	3.793	6.961	32.039	1.517
Blanco, NM	0.712	2.279	0.285	0.712	1.282	10.114	0.285
Blanding, UT	1.878	0.567	0.437	0.34	0.599	2.672	0.13
Bloomfield, NM	11.705	6.786	1.285	3.04	3.621	41.556	0.594
Bluff, UT	11.772	6.746	2.778	3.307	5.291	19.577	0.794
Burnham Chapter, NM	3.409	2.727	1.736	1.364	4.091	11.53	0.434
Counselor, NM	1.718	3.113	0.322	1.235	2.576	16.318	0.107
Cudei, NM	7.734	2.486	0.502	1.733	1.808	9.391	0.377
Farmington, NM	9.056	3.917	0.554	2.244	2.472	21.075	0.732
Fruitland, NM	7.768	3.369	0.792	1.678	2.976	15.517	0.673
Hogback Chapter, NM	13.156	4.019	0.582	2.112	3.222	18.252	0.603
Huerfano, NM	3.992	4.128	0.568	1.49	2.115	26.044	0.219
Kimbeto, NM	5.566	3.597	7.193	0.428	1.97	11.903	0
Kirtland, NM	7.483	2.631	0.274	1.384	1.8	12.785	0.548
La Plata, NM	2.614	2.179	0	0.436	1.598	9.586	0.726
Little Water, NM	5.487	4.05	1.045	1.934	4.572	16.931	0.575
Lybrook, NM	2.536	2.254	0.564	0.926	1.087	16.747	0.04
Mexican Water, AZ	3.029	1.597	0.092	0.551	1.303	6.297	0.349
Montezuma Creek, UT	6.358	3.894	0.548	2.282	3.255	15.606	1.004
Nageezi, NM	6.887	8.326	1.561	2.342	3.153	53.994	0.275
Nenahnezad, NM	6.612	3.808	0.335	2.051	2.553	14.438	0.398
Newcomb, NM	8.742	4.455	0.735	2.774	4.308	20.741	0.757
Ojo Amarillo, NM	7.146	3.404	0.299	1.833	1.553	16.48	0.58
Red Mesa, AZ	12.41	6.441	1.804	3.178	7.558	24.219	1.031
Red Valley Chapter, NM	29.496	8.789	1.565	5.393	9.422	39.551	1.898
Sanostee, NM	5.482	3.727	0.621	1.662	3.447	19.955	0.691
Sheep Springs, NM	4.387	2.063	0.316	0.744	2.565	11.934	0.669
Shiprock, NM	16.762	5.975	1.222	3.652	4.876	28.011	1.204
Teec Nos Pos, AZ	10.285	5.027	0.924	3.525	3.917	23.343	1.167
Toadlena, NM	5.497	6	1.106	1.676	2.447	24.434	0.503
Tocito, NM	2.545	2.219	0	0.392	0.587	9.659	0.131
Towaoc, CO	0.787	0.801	0.129	0.429	0.858	3.618	0.315
Two Grey Hills, NM	9.885	5.95	1.141	2.356	4.226	32.228	0.461
Waterflow, NM	2.451	1.03	0.154	0	0.947	5.282	0.178
White Mesa, UT	0.163	0.109	0	0	0.163	1.144	0

**Table 3:** Selected results for trace metal concentrations (*μ*g/m^3^) from ambient air samples collected in 2005. Zero values indicate that the element, if present, was below the detection limits.

Element	Nav1	Nav10	Nav22	Nav16	Nav50	Nav7	Nav44	Nav38	Nav11	Nav34	Nav25	Nav26	health levels
arsenic (As)	0.005	0.012	0.014	0.021	0.028	0.001	0.004	0.040	0.001	0.005	0.007	0.006	10^a^
beryllium (Be)	0	0	0	0	0	0	0	0	0	0	0	0	0.01^b^
chromium (Cr)	0.003	0.013	0.014	0.001	0	0.007	0	0.014	0	0.007	0	0	500^c^
cadmium (Cd)	0	0	0	0	0	0	0	0	0	0	0	0	100^a^
lead (Pb)	0	0.002	0.003	0.005	0.004	0.002	0.002	0.007	0.001	0.001	0.002	0.003	1.5^d^
manganese (Mn)	0.001	0.008	0.005	0.007	0.007	0.003	0.002	0.026	0.003	0.005	0.007	0.014	5000^a^
mercury (Hg)	0	0	0	0.001	0	0	0	0.001	0	0.001	0.002	0.001	100^a^
nickel (Ni)	0	0.003	0	0	0	0.008	0.001	0.018	0	0.001	0.003	0	1000^a^
selenium (Se)	0.001	0.007	0.010	0.007	0.009	0.004	0.003	0.041	0.002	0.004	0.004	0.008	200^a^
antimony (Sb)	0	0.001	0.001	0.001	0.002	0.001	0.002	0.001	0	0	0.002	0.002	500^a^
cobalt (Co)	0	0	0	0	0	0	0	0	0	0	0	0	100^a^

(a) Agency for Toxic Substances and Disease Registry—8 hour work day, 40 hour work week (http://www.atsdr.cdc.gov/tfacts4.html).

(b) Agency for Toxic Substances and Disease Registry—30 day period (http://www.atsdr.cdc.gov/tfacts4.html).

(c) National Institute for Occupational Safety and Health—time-weighted average (10 hours)

(http://www.atsdr.cdc.gov/HEC/CSEM/chromium/standards_regulations.html).

(d) U.S. EPA, NAAQS—quarterly average (http://www.epa.gov/air/criteria.html).

**Table 4:** Trace element concentrations (ppm) from coal samples taken from the Four Corners mine (BHP), Black Mesa, and five residences in or near Shiprock, New Mexico. Mean concentrations of trace metals in US coals [23] are in the final column for comparison.

element	BHP	Black Mesa	Nav51	Nav56	Nav69	Nav78	Nav151	mean US coal
arsenic (As)	0.272	0.603	0.481	0.571	0.329	0.48	0.363	24.0
beryllium (Be)	0.807	0.103	1.13	0.101	0.744	0.465	0.559	2.20
chromium (Cr)	3.20	2.26	9.83	3.66	2.71	2.33	4.05	15.0
cadmium (Cd)	0.021	0.007	0.069	0.009	0.015	0.013	0.018	0.470
lead (Pb)	9.76	0.829	19.7^a^	0.730	5.46	1.08	4.14	11.0
manganese (Mn)	10.0	1.93	10.0	3.60	6.93	5.13	9.56	43.0
mercury (Hg)	0.016	0.026	0.027	0.029	0.015	0.02	0.120	0.170
nickel (Ni)	2.21	2.21	3.47	2.86	2.09	1.60	3.07	14.0
selenium (Se)	1.70	0.770	2.40	1.40	1.70	1.30	0.650	2.80
antimony (Sb)	0.512	0.058	0.496	0.116	0.304	0.234	0.318	1.20
cobalt (Co)	2.57	0.568	5.13	0.74	2.58	0.973	1.78	15.0

a. Only one element (lead) in one sample (Nav51) exceeded the U.S. average values in coal for these hazardous air pollutants.

**Table 5:** Semiquantitative results of polycyclic aromatic hydrocarbon (PAH) concentration (ng/m^3^) in 24-hour PM_2.5_ air samples.

	NAV78 winter indoor	NAV151 winter indoor	NAV78 winter outdoor	NAV151 winter outdoor	NAV151 summer indoor
1 methylnaphthalene	1.378	1.803	1.105	1.721	0.988
1 methylphenanthrene	0.000	0.000	0.873	3.588	0.000
2 methylnaphthalene	0.747	0.936	1.017	0.929	0.591
2,3,5-trimethylnaphthalene	0.000	0.000	0.000	0.000	0.000
2,6-dimethylnaphthalene	0.344	0.000	0.430	0.000	0.000
3,6-dimethylphenanthrene	0.000	0.000	0.000	0.565	0.000
acenaphthene	0.000	0.000	0.000	0.000	0.000
acenaphthylene	0.000	0.000	0.000	1.345	0.000
anthracene	0.000	0.071	0.000	1.773	0.000
benzo[a]anthracene	4.346	3.760	5.040	28.968	0.000
benzo[a]pyrene	5.223	21.813	4.693	26.504	3.443
benzo[b] fluoranthene	22.654	31.709	8.021	42.211	9.562
benzo[e]pyrene	14.312	33.987	5.694	24.674	14.396
benzo[ghi]perylene	19.706	116.106	5.684	25.986	81.793
benzo[k] fluoranthene	22.500	32.723	8.782	41.669	7.067
biphenyl	0.000	0.762	0.337	0.000	0.000
chrysene	8.829	5.676	7.505	39.672	0.000
dibenzo[ah]anthracene	5.078	8.737	6.757	7.699	0.000
dibenzothiophene	0.000	0.000	0.000	0.000	0.000
fluoranthene	2.818	4.494	3.746	23.894	1.444
fluorene	0.283	0.000	0.000	0.699	0.000
indeno[123cd]pyrene	27.720	81.681	7.143	36.943	38.791
naphthalene	2.322	4.399	1.395	2.851	2.010
perylene	0.000	5.377	2.926	7.058	0.000
phenanthrene	2.860	5.068	2.749	12.368	0.000
pyrene	1.667	5.681	4.004	25.749	1.713
total ng	142.786	364.781	77.902	356.866	161.797
average ng	5.492	14.030	2.996	13.726	6.223
avg ng/m^3^	0.171	0.436	0.093	0.427	0.193
